# Quantification of Phenolic Compounds by HPLC/DAD and Evaluation of the Antioxidant, Antileishmanial, and Cytotoxic Activities of Ethanolic Extracts from the Leaves and Bark of *Sarcomphalus joazeiro* (Mart.)

**DOI:** 10.3390/plants14111733

**Published:** 2025-06-05

**Authors:** Natália Kelly Gomes de Carvalho, Débora Odília Duarte Leite, Aracélio Viana Colares, Fernando Almeida Souza, Kátia da Silva Calabrese, Gerson Javier Torres Salazar, Joice Barbosa do Nascimento, Mariana Pereira da Silva, Fabiola Fernandes Galvão Rodrigues, José Galberto Martins da Costa

**Affiliations:** 1 Northeast Biotechnology Network—RENORBIO, State University of Ceará, Av. Dr. Silas Munguba, 1700—Campus do Itaperi, Fortaleza 60714-903, CE, Brazil; nataliakellygc@gmail.com; 2 Department of Biological Chemistry, Natural Products Research Laboratory, Universidade Regional do Cariri, Rua Coronel Antônio Luíz, 1161—Pimenta, Crato 63105-010, CE, Brazil; biodeboraleite@yahoo.com.br (D.O.D.L.); timotygertor@yahoo.com (G.J.T.S.); joicenascimento2010@live.com (J.B.d.N.); mariana.pereira@urca.br (M.P.d.S.); fabiolafer@gmail.com (F.F.G.R.); 3 Biomedicine Course, Doctor Leão Sampaio University Center, Av. Leão Sampaio, 400—Lagoa Seca, Juazeiro do Norte 63040-000, CE, Brazil; avcolares@gmail.com; 4 Protozoology Laboratory, Instituto Oswaldo Cruz, Fiocruz, Rio de Janeiro 21040-900, RJ, Brazil; fernandosouza@professor.uema.br (F.A.S.); calabrese@ioc.fiocruz.br (K.d.S.C.); 5 Protozoology Laboratory, State University of Maranhão, Av. Lourenço Vieira da Silva, n.º 1000, Jardim São Cristóvão, São Luís 65055-310, MA, Brazil

**Keywords:** *Sarcomphalus joazeiro*, antioxidant, antiparasitic, cytotoxic

## Abstract

*Sarcomphalus joazeiro* (Mart.) is a promising candidate for the formulation of new therapies against parasitic infections. This study aimed to quantify the content of phenolic compounds and evaluate the antioxidant, antileishmanial, and cytotoxic potential of ethanolic extracts of the leaves (EELSJ) and bark (EEBSJ) of *S. joazeiro*. Quantification of phenolic acids (caffeic acid, *p*-coumaric acid, ferulic acid, cinnamic acid) and flavonoids (naringenin, pinocembrin, and apigenin) was performed by high-performance liquid chromatography with a diode array detector (HPLC-DAD). The extracts were subjected to antioxidant assays, including Fe^3+^ reduction, Fe^2+^ chelation, and inhibition of oxidative degradation of deoxyribose (2-DR). The antileishmanial activity was evaluated against promastigote forms of *Leishmania amazonensis*, while cytotoxicity was assessed in J774.G8 macrophages. Among the biological effects evaluated, EELSJ showed potent hydroxyl radical (•OH) scavenging activity, with IC_50_ < 10 µg/mL, which potentially correlates with its phenolic acid and flavonoid content (0.7066 mg/g). In comparison, EEBSJ showed a lower phenolic content (0.197 mg/g) and demonstrated Fe^2+^ chelating activity (IC_50_ = 14.96 ± 0.0477 µg/mL). EELSJ also exhibited antileishmanial activity against *L. amazonensis* (IC_50_ = 246.20 µg/mL), with low cytotoxicity (CC_50_ = 343.3 µg/mL; SI = 1.39), whereas EEBSJ showed minimal antileishmanial effect and marked cytotoxicity toward J774.G8 macrophages (CC_50_ = 5.866 µg/mL). The leaves of *S. joazeiro* stand out as the most promising plant organ for future investigations. Future studies should focus on investigating their action mechanisms in more detail.

## 1. Introduction

Leishmaniases are neglected tropical diseases that pose a significant burden on public health. In 2021, Brazil reported the highest number of annual infections among South American countries, with 15,023 confirmed cases. In leishmanial infections, the etiological agent *Leishmania amazonensis* can cause severe cutaneous lesions and, in some instances, more serious manifestations such as diffuse cutaneous leishmaniasis, which may be fatal under certain conditions [1,2]. Conventional treatment options are primarily limited to pentavalent antimonials, amphotericin B, and pentamidine, among others. These drugs are often associated with parasite resistance and considerable toxicity in humans [3].

Oxidative stress plays a crucial role in the host’s defense mechanisms against *Leishmania*. Typically, the immune system generates reactive oxygen species (ROS) to combat invading pathogens. Although effective in eliminating microorganisms, excessive ROS levels can lead to host tissue damage, exacerbate inflammation, and contribute to disease progression [4]. In this context, the search for alternative therapies, particularly natural products capable of modulating oxidative stress, represents a promising advance in the fight against parasitic infections [5].

Plants produce a wide range of secondary metabolites, among which flavonoids and phenolic acids are the most prominent. The literature indicates that these compounds can neutralize free radicals due to their ability to donate electrons and protons, thus exhibiting antioxidant properties [6]. Moreover, they have demonstrated inhibitory effects on protozoan parasites, including *Leishmania* spp., through mechanisms such as oxidative stress induction and enzymatic regulation [7].

Brazil, with its vast territory and diverse climatic zones, offers considerable ecological variability, making it an important resource for the biotechnological exploration of natural products [8]. The Caatinga, for instance, is the only exclusively Brazilian biome. Located in the semi-arid Northeast, the region is characterized by a tropical semi-arid climate and a high degree of endemism, which supports its extensive biodiversity [9].

Among its endemic species, *Ziziphus joazeiro* Mart., recently reclassified by World Flora Online (2025) as *Sarcomphalus joazeiro* Mart. (Rhamnaceae), is popularly known as “joazeiro”, “juá-babão”, “juazeiro”, and “juá”. It is traditionally used in folk medicine as an oral antiseptic, antifungal, bactericide, and expectorant, and also for the treatment of gastric ulcers [10,11]. Recently, Carvalho et al. (2025), using ultra-performance liquid chromatography coupled with mass spectrometry (UHPLC-MS), identified several important flavonoids (rutin, kaempferol, isorhamnetin, and quercetin) and saponins (jujubasaponins I and II) in *S. joazeiro*, which are considered promising candidates due to their antioxidant and antiparasitic potential [12].

Given its biological potential, *S. joazeiro* is suggested to possess antiparasitic properties. Therefore, the present study aimed to quantify the phenolic acid and flavonoid contents and to evaluate the antioxidant, antileishmanial, and cytotoxic activities of leaf and bark extracts from *S. joazeiro*.

## 2. Results

### 2.1. Quantification of Phenolic Acids by HPLC/DAD

**Figure 1:**
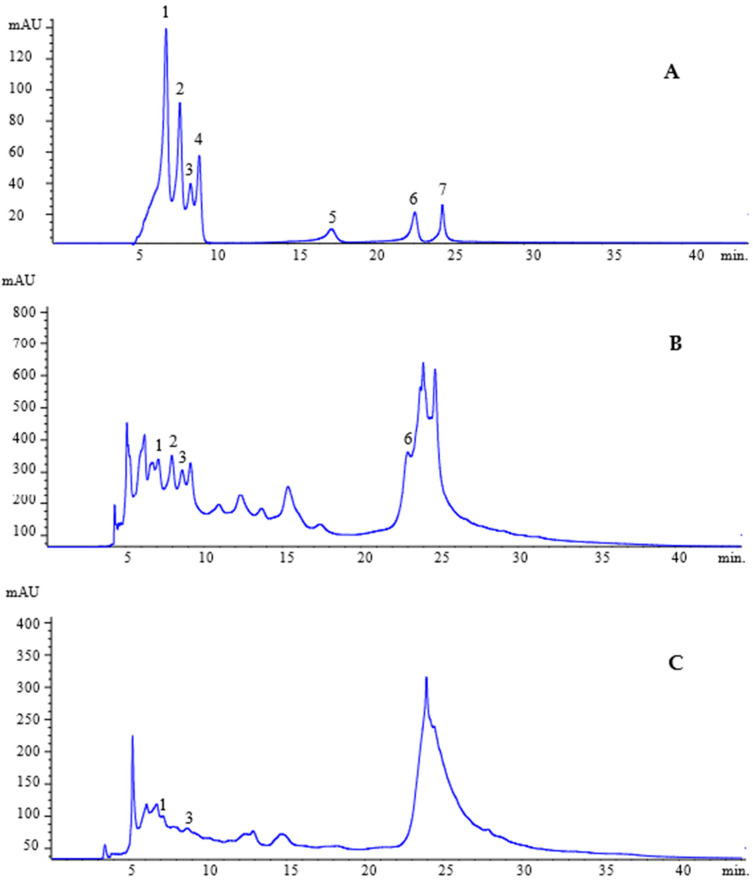
HPLC profile of phenolic acids and flavonoids standards. (**A**) Caffeic acid (tR = 7.1 min, peak 1), *p*-coumaric acid (tR = 8.0 min, peak 2), ferulic acid (tR = 8.7 min, peak 3), cinnamic acid (tR = 9.2 min, peak 4), naringenin (tR = 17.7 min, peak 5), pinocembrin (tR = 23.0 min, peak 6), and apigenin (tR = 24.7 min, peak 7). (**B**) HPLC/DAD profile of EELSJ: caffeic acid (peak 1), *p*-coumaric acid (peak 2), ferulic acid (peak 3), pinocembrin (peak 6) (**C**) HPLC/DAD profile of EEBSJ: Caffeic acid (peak 1), ferulic acid (peak 3).

Chemical analysis by HPLC-DAD revealed a total of 0.7066 mg/g of phenolic acids and flavonoids in the leaf extract (EELSJ). These were distributed among four compounds: the phenolic acids, namely caffeic acid (0.0456 ± 0.000057 mg/g), *p*-coumaric acid (0.0369 ± 0.017609 mg/g), and ferulic acid (0.2313 ± 0.002367 mg/g), and the flavonoid pinocembrin (0.3928 ± 0.007621 mg/g) (Table 1; Figure 1A,B).

In contrast, the bark extract (EEBSJ) contained a total of 0.197 mg/g of phenolic acids, distributed across two compounds: caffeic acid (0.0470 ± 0.020785 mg/g) and ferulic acid (0.150 ± 0.008660 mg/g) (Table 1; Figure 1A,C).

**Figure 2:**
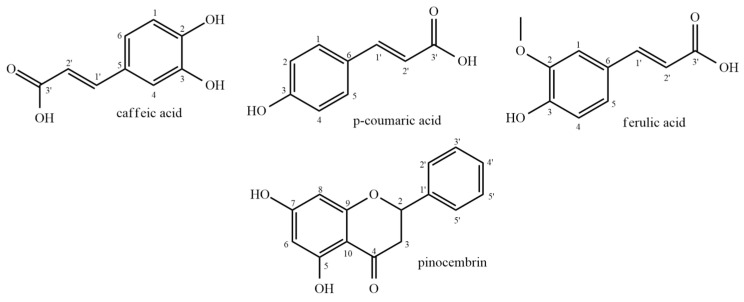
Chemical structure of the compounds quantified by HPLC/DAD in the ethanolic extracts of leaves (EELSJ) and bark (EEBSJ) of *S. joazeiro.* Source: The same author (2025). Structures were drawn using ChemDraw Professional version 12.0.2.1076 (PerkinElmer Inc., Waltham, MA, USA).

The chemical structures of the compounds quantified in *S. joazeiro* were drawn using ChemDraw Professional version 12.0.2.1076 (PerkinElmer Inc., Waltham, MA, USA) (Figure 2).

### 2.2. Antioxidant Assay

#### 2.2.1. Fe^2+^ Chelating Activity and Fe^3+^ Reducing Power

**Figure 3:**
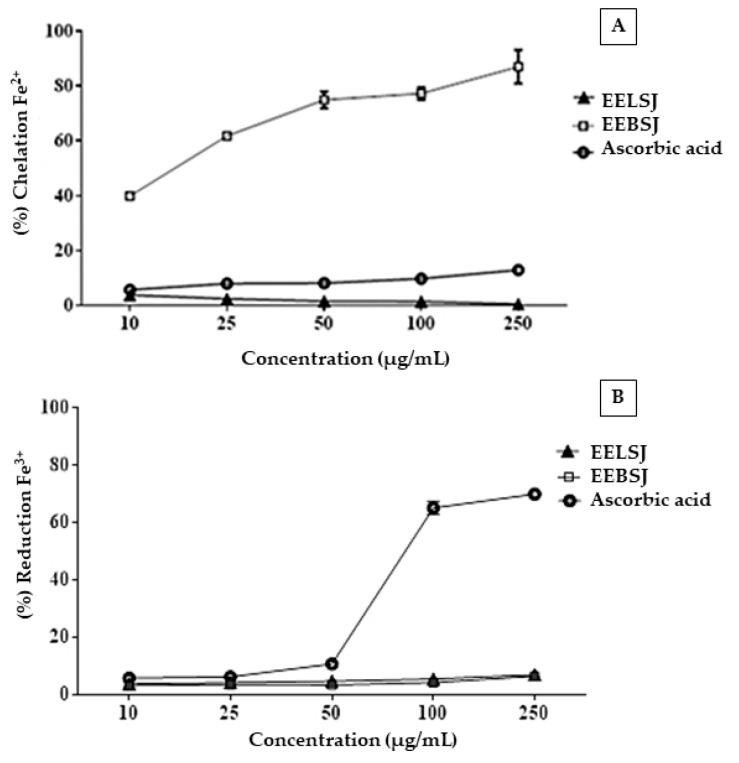
(**A**) Fe^2+^ chelating potential of the ethanolic extracts from the bark (EEBSJ) and leaves (EELSJ) of *S. joazeiro*, and the antioxidant standard ascorbic acid, assessed by the *o*-phenanthroline assay. (**B**) Fe^3+^ reducing potential of the ethanolic extracts from the bark (EEBSJ) and leaves (EELSJ) of *S. joazeiro*, and the antioxidant standard ascorbic acid, assessed by the *o*-phenanthroline assay. Values are expressed as means ± SEM (n = 4) and were analyzed by one-way ANOVA followed by Tukey’s multiple comparisons test, using a single pooled variance: significant difference (*p* < 0.05).

As shown in Figure 3A, EEBSJ exhibited significant Fe^2+^ chelating activity (*p* < 0.0477), reaching a maximum chelation rate of 90.54% at a concentration of 250 µg/mL. The mean inhibitory concentration (IC_50_) was determined to be 14.96 ± 0.0477 µg/mL, indicating a strong interaction with the metal ion. In contrast, the ethanolic extract of the leaves (EELSJ) and the antioxidant standard (ascorbic acid) showed no detectable chelating activity under the experimental conditions, with chelation percentages significantly lower than those exhibited by EEBSJ.

In the Fe^3+^ reduction assay (Figure 3B), both extracts presented values below 50%, making it impossible to calculate the IC_50_. The antioxidant standard (ascorbic acid) presented an IC_50_ of 70.59 ± 0.0510 µg/mL.

#### 2.2.2. Deoxyribose Oxidative Degradation Assay

**Figure 4:**
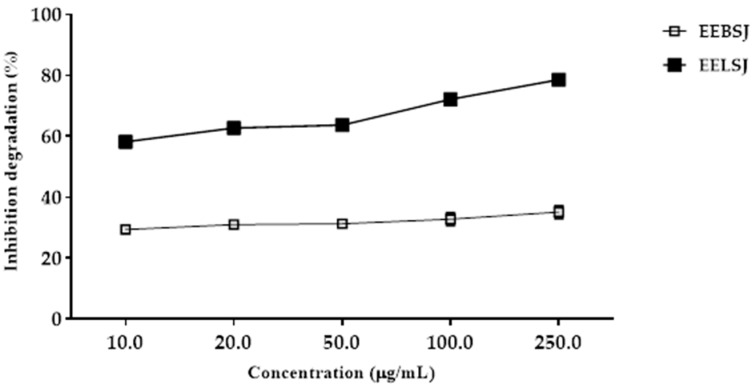
Inhibition degradation assay of deoxyribose (2-DR) associated with the ethanolic extracts from the bark (EEBSJ) and leaves (EELSJ) of *S. joazeiro*. Values are expressed as means ± SEM (n = 4) and were analyzed by one-way ANOVA followed by Tukey’s multiple comparisons test, using a single pooled variance: significant difference (*p* < 0.05).

The sequestration potential of •OH was evaluated using the oxidative degradation assay of 2-deoxyribose (2-DR), quantifying its byproduct, malondialdehyde (MDA) [13]. EELSJ was able to inhibit the degradation of 2-DR, with an IC_50_ value of <10 µg/mL. On the other hand, at the concentrations tested, it was not possible to calculate the IC_50_ of EEBSJ (Figure 4).

### 2.3. Antileishmanial Activity

**Figure 5:**
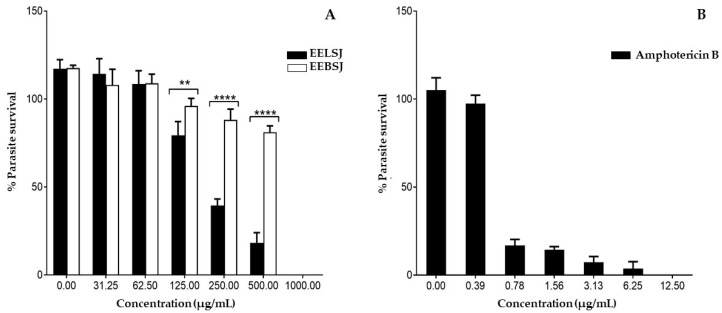
Antileishmanial activity of ethanolic extracts of leaves (EELSJ) and bark (EEBSJ) of *Sarcomphalus joazeiro* (**A**), and amphotericin B (**B**), showing the percentage of survival of promastigotes of *Leishmania amazonensis* after 24 h of treatment at different concentrations (µg/mL). Small increases above 100% reflect normal biological and instrumental variability and do not indicate increased parasite burden. Data represent mean ± standard deviation of three independent experiments performed in triplicate. ** *p* = 0.0086; **** *p* < 0.0001 by two-way ANOVA followed by Sidak’s multiple comparisons test.

The antileishmanial activity of ethanolic extracts of *S. joazeiro* was evaluated against the promastigote form of *L. amazonensis* (Figure 5A, Table 2). Both extracts promoted a significant and dose-dependent reduction in parasites. However, the EELSJ showed significantly higher leishmanicidal activity compared to the EEBSJ, especially at concentrations of 125, 250, and 500 µg/mL (*p* = 0.0086; **** *p* < 0.0001). EELSJ exhibited an IC_50_ value of 246.20 ± 0.089 µg/mL, while EEBSJ did not show significant activity within the concentrations tested, with an estimated IC_50_ of 880.7 ± 0.062 µg/mL, being more than three times the IC_50_ for EELSJ, providing low efficacy. Amphotericin B, used as a positive control, demonstrated high antileishmanial potency, with an IC_50_ of 0.54 ± 0.122 µg/mL (Figure 5B, Table 2).

### 2.4. Cytotoxicity

The absence of toxicity of natural compounds to host cells is a crucial factor in the therapeutic approach against *L. amazonensis* parasites. In this context, J774G8 macrophages were tested to determine cytotoxicity. The selectivity index (SI), which is the ratio between the 50% cytotoxic concentration (CC_50_) and the IC_50_ value for the tested parasite, was compared for both extracts.

EELSJ did not demonstrate significant cytotoxicity against J774G8 macrophages, with CC_50_ = 343.3 ± 0.98 µg/mL and SI > 1. In contrast, EEBSJ exhibited high cytotoxicity against J774G8 macrophages, with a CC_50_ of 5.866 ± 1.027 µg/mL (Table 2) and SI < 1, indicating greater toxicity to host cells than to the parasite, making it unsuitable for therapeutic use.

## 3. Discussion

This is the first study to quantify phenolic acids (caffeic, *p*-coumaric, ferulic, and cinnamic) and flavonoids (naringenin, pinocembrin, and apigenin) in the chemical composition of the leaves and bark of *S. joazeiro*. It is also the first investigation to evaluate the antioxidant potential of this species using Fe^2+^ chelation, Fe^3+^ reduction, and deoxyribose degradation inhibition assays, as well as to assess its antileishmanial activity against *L. amazonensis* promastigotes and cytotoxicity in J774.G8 macrophages. These findings significantly expand the current knowledge of the bioactive properties of *S. joazeiro*, with emphasis on its antioxidant and antiparasitic effects.

Previous studies confirmed the presence of flavonoids and saponins in *S. joazeiro* through ultra-high-performance liquid chromatography coupled with mass spectrometry (UHPLC-MS) [11]. More recently, Carvalho et al. (2025) identified flavonoids (rutin, kaempferol, isorhamnetin, and quercetin) and saponins (jujubasaponin I and II) in the leaf extract of the species [12].

Earlier, Brito et al. (2015) [14] quantified phenolic acids (gallic, chlorogenic, caffeic, and ellagic) and flavonoids (catechin, epicatechin, rutin, isoquercitrin, quercitrin, quercetin, and kaempferol) in *S. joazeiro* leaves, reporting concentrations of 183.136 mg/g and 7.37 mg/g of extract, respectively. In contrast, the present study performed a partial quantification by HPLC-DAD, yielding 0.7066 mg/g of phenolic acids and flavonoids in EELSJ, a significantly lower value. This difference may be attributed to the use of specific phenolic standards rather than total phenolic and flavonoid quantification, in addition to seasonal factors such as the collection period and location, as well as extraction methods, which are known to influence secondary metabolite production [14,15].

On the other hand, the bark extract is well documented in the literature for its saponin content. Andrade et al. (2019) and Silva et al. (2024) identified several saponins, including jujuboside B, jujubasaponin III, ziziphin, jujubasaponin IV, jujuboside II, and zizyphus saponin I. The abundant presence of these compounds may explain the low levels of flavonoids and phenolic acids in EEBSJ, likely due to metabolic pathway shifts favoring triterpenoid saponin biosynthesis [11,16].

Despite its lower content of phenolic acids (0.197 mg/g), the EEBSJ exhibited higher Fe^2+^-chelating activity (Figure 3A). This effect may be attributed to the substantial amount of glycosylated saponins present in its chemical composition [16], whose triterpenoid structures contain hydroxyl (-OH) groups and regions of the pentacyclic core that likely favor the formation of micelles with soap-like properties. These micelles potentially exhibit high electron density in localized areas, promoting electrostatic interactions between these molecular sites and Fe^2+^ ions, ultimately resulting in the formation of stable chelates. In this interaction, Fe^2+^ ions become encapsulated by the large saponin molecules, rendering them unavailable to participate in reactions involving reactive oxygen species (ROS) [16,17,18].

In contrast, the EELSJ displayed limited chelating activity, similar to that of ascorbic acid (used as a reference antioxidant), which may be primarily attributed to the low concentrations of constituents widely recognized for their antioxidant and chelating potential, including, but not limited to, phenolic compounds [19,20].

The absence of Fe^3+^-reducing activity observed in both extracts can be attributed to the low concentrations of reducing compounds, which were insufficient to produce a detectable effect in the assay (Figure 3B, Table 1). At high concentrations, some compounds may exhibit pro-oxidant behavior, as described by Brito et al. (2015) [14], who reported an IC_50_ of 870 µg/mL for the leaf extract of *S. joazeiro* and suggested a potential pro-oxidant effect of caffeic acid. Furthermore, the saponins present in EEBSJ may not contribute to Fe^3+^ reduction due to the absence of conjugated electron systems and the predominance of glycosidic and triterpenoid structures, which possess limited electron-donating capacity and, consequently, low reducing potential [14,21].

In the deoxyribose degradation inhibition assay, EELSJ showed an IC_50_ < 10 µg/mL, while EEBSJ did not exhibit a consistent response that would allow for IC_50_ determination (Figure 4). The presence of phenolic acids and flavonoids in EELSJ, such as caffeic, *p*-coumaric, and ferulic acids, as well as pinocembrin (Table 1), may potentiate the antioxidant effect through synergistic interactions, even at individually low concentrations, thereby enhancing the protection of deoxyribose.

From a chemical perspective, these compounds may act as proton donors and stabilize free radicals through resonance. The number and position of hydroxyl groups on the aromatic ring, along with the presence of electron-donating substituents, significantly influence this stability [22,23].

For instance, the para-hydroxyl group on caffeic acid enhances its radical-stabilizing effect, making it a more potent antioxidant. In *p*-coumaric acid, the conjugated double bond allows for electron delocalization and radical stabilization. In ferulic acid, the methoxy group and ortho-hydroxyl group further contribute to the stabilization of the phenoxyl radical. Pinocembrin features hydroxyl groups at positions 5 and 7 on ring A, which facilitate electron or proton donation to neutralize free radicals such as hydroxyl radicals [13].

The significant antileishmanial activity of EELSJ may be partially attributed to its high antioxidant capacity, as evidenced by its effective neutralization of hydroxyl radicals (•OH) in the deoxyribose degradation inhibition assay (Table 2, Figure 5). Hydroxyl radicals are among the most aggressive ROS, capable of oxidizing lipids, proteins, and nucleic acids. In the context of *Leishmania* infection, ROS also play critical roles in redox signaling pathways that are essential for parasite adaptation and virulence [5].

This effect may be due, though not exclusively, to the synergistic action of flavonoids and phenolic acids identified in the extract (Table 1) [12]. These compounds have been associated with disruption of redox homeostasis in *Leishmania*, promoting mitochondrial dysfunction, inhibition of ergosterol biosynthesis, and oxidative damage, ultimately leading to parasite cell death [13,24]. Among the phenolic compounds quantified in *S. joazeiro*, caffeic acid stands out due to its proven antileishmanial activity against intracellular amastigote forms in RAW264.7 macrophages [25], reinforcing its potential contribution to the effects observed in this study.

Several molecular mechanisms have been proposed to explain these effects, including the following: (a) mitochondrial membrane potential depolarization, impairing ATP generation; (b) inhibition of pteridine reductase 1, an enzyme essential for folate metabolism in the parasite; and (c) inhibition of farnesyl diphosphate synthase, which is involved in ergosterol biosynthesis, a key component of the parasite’s plasma membrane [7,26]. These mechanisms converge to reduce parasite viability and support the antileishmanial effects observed.

In comparison, Brito et al. (2015) [14] evaluated the antileishmanial activity of *S. joazeiro* leaf extract and reported IC_50_ values exceeding 5.000 µg/mL for *L. braziliensis* and 693.67 µg/mL for *L. infantum*, indicating low efficacy against the parasite. These values are considerably higher than those observed in the present study, in which EELSJ exhibited an IC_50_ of 246.20 µg/mL against *L. amazonensis* promastigotes, suggesting a greater potential to inhibit parasite growth. This difference can be attributed to factors such as differences in the Leishmania species evaluated and the variation in the profile of secondary metabolites found.

The literature supports these findings, indicating that species of the Rhamnaceae family exhibit significant leishmanicidal activity, often attributed to the presence of phenolic acids and flavonoids. Albalawi (2021) demonstrated that the methanolic extract of *Z. spina-christi* significantly reduced (*p* < 0.001) the viability of *L. major* amastigotes, with an IC_50_ of 54.6 µg/mL. This activity was attributed to the presence of flavonoids, tannins, saponins, and glycosides. Similarly, Hammi et al. (2022) reported that dichloromethane and methanolic extracts of *Z. lotus* exhibited relevant activity against *L. major* (IC_50_ = 20.55 ± 0.34 µg/mL and 226.64 ± 2.46 µg/mL, respectively) and *L. infantum* (IC_50_ = 15.37 ± 0.17 µg/mL and 285.67 ± 1.77 µg/mL, respectively). These effects were associated with flavonoids such as catechin, rutin, and luteolin-7-O-glucoside, identified by HPLC [18,27].

EEBSJ demonstrated greater cytotoxic potential against J774.G8 macrophages (Table 2). Recent studies suggest that the cytotoxicity of saponins may vary depending on their chemical structure, with an inverse correlation between toxicity and the number of sugar moieties, which affects saponin hydrophobicity [27].

Similar findings were reported by Faria et al. (2021), who examined *Montrichardia linifera* extracts rich in saponins. These extracts showed significant cytotoxicity against J774.G8 macrophages, with CC_50_ values of 54.82 µg/mL (stem) and 26.95 µg/mL (leaves) [28]. Although saponins possess notable biological activity, their therapeutic use may be limited by toxicity, underscoring the need for further studies on structural modifications aimed at reducing adverse effects.

Given the increasing resistance to antimonial drugs and the adverse effects associated with conventional treatments, the leaf extract (EELSJ) of *S. joazeiro* may represent a promising phytotherapeutic alternative for leishmaniasis treatment. Further studies are necessary to validate these findings in more complex biological systems, to confirm therapeutic efficacy, elucidate molecular mechanisms, and evaluate the safety of isolated compounds.

## 4. Materials and Methods

### 4.1. Sample Collection and Preparation

Bark (900 g) and leaves (900 g) were collected in Tabocas, a rural area in the municipality of Exu, Pernambuco, Brazil. The voucher specimen was deposited in the Herbarium Dárdano de Andrade Lima of the Regional University of Cariri—URCA, under registration number 13,346, and was identified as *Sarcomphalus joazeiro* Mart. The plant material was macerated in hexane (Sigma-Aldrich, St. Louis, MO, USA) for 72 h at room temperature to remove fat. After the elimination of the solvent and complete drying, the plant materials were subjected to extraction in absolute ethanol (Sigma-Aldrich) to obtain the crude extract. The organic solvents were removed using a rotary evaporator under reduced pressure. The hexane extracts were stored, and the ethanolic extracts used in this study presented the following extraction efficiencies (*w*/*w*): ethanolic extract of the bark of *S. joazeiro* (EEBSJ, 4.50%) and ethanolic extract of the leaves (EELSJ, 4.70%).

### 4.2. Quantification of Phenolic Acids and Flavonoids by HPLC/DAD

#### 4.2.1. Preparation of Standards

Standard solutions for phenolic acids and flavonoids (Sigma-Aldrich), namely caffeic acid, *p*-coumaric acid, ferulic acid, cinnamic acid, naringenin, pinocembrin, and apigenin, were prepared in HPLC-grade methanol (Agilent Technologies, Waldbronn, Germany ) to produce stock solutions at a concentration of 1000 ppm. These were subsequently diluted to concentrations ranging from 1 to 50 ppm. The absorbances of the 20 ppm standards were measured at wavelengths from 190 to 400 nm in order to determine the maximum detection wavelength for HPLC-DAD measurements.

Calibration curve for caffeic acid: y = 331.13x − 94.735 (r^2^ = 0.9999); *p*-coumaric acid: y = 449.98x − 72.528 (r^2^ = 0.9971); ferulic acid: y= 185.3x −675.79 (r^2^ = 0.9887); cinnamic acid: y = 236.74x − 188.67 (r^2^ = 0.9991); naringenin: y= 151.64x + 8.5796 (r^2^ = 0.9994); pinocembrin: y = 177.66x − 390.72 (r^2^ = 0.9955); and apigenin: y = 164.9x − 688.69 (r^2^ = 0.9963).

#### 4.2.2. HPLC/DAD Analysis

The quantification of phenolic acids and flavonoids was performed by high-performance liquid chromatography (HPLC) using an Agilent 1260 system (Agilent Technologies, Germany) equipped with a diode array detector (DAD) and an autosampler. Separation was carried out on a C18 column (250 mm × 4.0 mm; 5 µm; Macherey-Nagel, Düren, Germany) using a gradient elution method with a mobile phase composed of solvent A (ultrapure water) and solvent B (methanol:acetonitrile, 60:40, *v*/*v*, HPLC grade), both acidified with 0.1% formic acid (Sigma-Aldrich).

The elution profile was as follows: 0–15 min, 15% B; 17 min, 40% B; 30 min, 30% B; 38 min, 15% B, maintained until 45 min. The mobile phase flow rate was set to 0.5 mL/min, and the injection volume was 20 µL. Detection was performed at different wavelengths according to the target compound: 310 nm for caffeic, p-coumaric, and ferulic acids; 290 nm for cinnamic acid, naringenin, and pinocembrin; and 340 nm for apigenin.

The extracts (EELSJ and EEBSJ) were dissolved in HPLC-grade methanol (30 mg/mL) and analyzed in triplicate at room temperature. All mobile phases, solutions, and samples were filtered through 0.22 µm pore size, 13 mm diameter membrane filters (Millipore) prior to analysis. Compound identification was based on comparison of retention times and UV-Vis spectra (190–400 nm) with those of reference standards.

Limits of detection (LOD) and quantification (LOQ) were calculated based on the standard deviation of the response (σ) and the slope of the calibration curve (S), using three independent analytical curves and applying the following formulas: LOD = 3.3σ/S and LOQ = 10σ/S.

### 4.3. Antioxidant Activity

#### 4.3.1. Fe^2+^ Chelating Activity and Fe^3+^ Reducing Power

EEBSJ and EELSJ were prepared from a stock concentration of 12.000 µg/mL diluted in PA ethanol (Sigma-Aldrich). Final concentrations of the extracts ranged from 10 to 250 µg/mL in the wells. For testing, 500 µL of each extract was mixed with 500 µL of in vitro Fe^2+^ ions using a 1.000 µM FeSO_4_ aqueous solution and 500 µL of in vitro Fe^3+^ ions using a 1.000 µM FeCl_3_ aqueous solution, under light and refrigeration conditions. Subsequently, 50 μL of the mixture was transferred to 96-well plates containing 250 μL of a tris-HCl buffer solution with *o*-phenanthroline at pH 7.4 ± 0.1 (Sigma-Aldrich). The blank was prepared by replacing the tris-HCl/phenanthroline mixture with Milli-Q water. The reaction time was 2.5 min, and the absorbance was measured using an ELISA microplate reader at 510 nm; the calculations performed to determine the percentage of chelation and reduction are illustrated in Equations (1) and (2), respectively [5]. Calibration curves of the standard (ascorbic acid) for the chelation of Fe^2+^ (y = 0.0197x + 7.553, R^2^ = 0.998) and reduction of Fe^3+^ (y = 0.1492x + 4.1788, R^2^ = 0.9942).

Calculation for percentage of Fe^2+^ chelation:

(1) $$\frac{[Abs_{control}de\;Fe^{2+}-(Abs_{sample}+Fe^{2+}-Abs_{white}+Fe^{2+})]\times 100\%}{Abs_{control}Fe^{2+}}$$

Calculation for percentage reduction of Fe^3+^

(2) $$\frac{100-[Abs_{control}de\;Fe^{2+}-(Abs_{sample}+Fe^{3+}-Abs_{white}+Fe^{3+})]\times 100\%}{Abs_{control}Fe^{2+}}$$

#### 4.3.2. Deoxyribose Oxidative Degradation Assay

The hydroxyl radical (-OH) is a highly frightening reactive oxygen species, capable of inducing significant damage to various cell structures, such as lipids, proteins, and DNA. In vivo, this radical can be generated from the interaction between superoxide radicals and transition metal ions, such as iron and copper, through the Haber–Weiss event [5].

To carry out the hydroxyl radical scavenging test, the EELSJ and EEBSJ extracts were prepared from a stock solution of 30.000 µg/mL diluted in PA grade ethanol (Sigma-Aldrich). The final concentrations tested ranged from 10 to 250 µg/mL. The reaction system consisted of 450 µL of potassium phosphate (7.5 mM, pH 7.4), 150 µL of aqueous deoxyribose solution (1.5 mM), 240 µL of hydrogen peroxide (H_2_O_2_, 0.8 mM), 240 µL of ferrous sulfate (FeSO_4_, 80 µM), and 320 µL of Milli-Q water. To this mixture, 100 µL of the extract solution was added. For the blank control, the occurrence was caused by the absence of deoxyribose.

The samples were incubated at 37 °C for 60 min. After this period, 750 µL of trichloroacetic acid (2.8%) and 750 µL of thiobarbituric acid (0.8%) were added, followed by a further incubation in a water bath at 100 °C for 20 min. The absorbance of the resulting solutions was measured in a spectrophotometer at 532 nm. All analyses were carried out in triplicate. The results were expressed as the percentage of inhibition of deoxyribose degradation in relation to the negative controls, according to Equation (3) [13].

Inhibition (%) = 100 − [((Abs_control_ − (Abs_sample_ − Abs_white_)) × 100)/Abs_control_] (3)

### 4.4. Antileishmanial and Cytotoxic Activity

#### 4.4.1. Parasites

Promastigote forms of *Leishmania amazonensis* (MHOM/BR/76/MA-76) were maintained at 26 °C in Schneider’s Insect Medium supplemented with 10% fetal bovine serum (Grand Island, NY, USA) and 100 U/mL penicillin (Gibco, Grand Island, NY, USA). To ensure the stability of the biological characteristics of the parasites, the cultures were used experimentally for up to ten in vitro passages [26].

#### 4.4.2. Cell Cultures

The macrophage J774.G8 line was cultured in RPMI 1640 medium (Sigma, USA) supplemented with 10% fetal bovine serum, penicillin (100 U/mL), and streptomycin (100 µg/mL) at 37 °C and 5% CO_2_.

#### 4.4.3. Activity Against Promastigote Forms

For antileishmanial and cytotoxicity assays, a stock solution in DMSO was prepared with extracts at 100 mg/mL, resulting in a maximum of 1% DMSO at the highest concentration evaluated.

Promastigote forms of *L. amazonensis* (10^6^ parasites/mL) from a 2–4-day-old culture were placed in 96-well plates in the presence of different concentrations of extracts (Sigma-Aldrich, ≥95% (GC); 31.25–1000 µg/mL, for both products) to a final volume of 100 µL per well for 24 h. Wells without parasites were used as blanks, and wells with only parasites were used as controls. After the treatment, the viability of parasites was evaluated by the tetrazolium-dye (MTT) colorimetric method modified by Mosmann [28]. MTT (5 mg/mL), a volume equal to 10% of the total, was added to each well. After 2 h, 100 µL of DMSO was added to dissolve the formazan. The absorbance was read on a spectrophotometer at a wavelength of 570 nm. The data was normalized according to Equation (4):

(4) $$\%survival=\frac{DO\;sample-DO\;blank}{DO\;control-DO\;blank}\times 100.$$

The results were used to calculate the IC_50_ (50% inhibition of parasite growth). Amphotericin B was used as the reference drug.

### 4.5. Cytotoxicity

The J774G8 macrophage cell line was cultured in RPMI 1640 medium (Sigma, St. Louis, MO, USA) supplemented with 10% fetal bovine serum, penicillin (100 U/mL), and streptomycin (100 µg/mL) at 37 °C and 5% CO_2_. Treatment, the viability assay with MTT, and CC_50_ determination (50% inhibition of cell growth, calculated similarly to IC_50_), were carried out as described in item Section 4.4.3 [26].

### 4.6. Statistical Analysis

Data were expressed as the mean ± SD and statistically analyzed by analysis of variance (ANOVA), followed by Tukey’s test (antioxidant assays) and Sidak’s test (antiparasitic assay). Analyses were performed using GraphPad Prism 8.0 software (GraphPad Software Inc., San Diego, CA, USA), and differences were considered significant when *p* < 0.05.

## 5. Conclusions

The leaf extract (EELSJ) presented a significantly higher concentration of phenolic compounds (0.706 mg/g) compared to the bark extract (EEBSJ) (0.197 mg/g), which was reflected in its superior performance in antioxidant and antileishmanial assays. Specifically, EELSJ showed hydroxyl radical scavenging capacity in the 2-deoxyribose degradation assay with an IC_50_ < 10 µg/mL, and exhibited antileishmanial activity (IC_50_ = 246.20 µg/mL) with low cytotoxicity toward J774.G8 macrophages. In contrast, EEBSJ exhibited Fe^2+^ chelating activity (IC_50_ = 14.96 ± 0.0477 µg/mL).

Overall, the leaves of *S. joazeiro* stand out as the most promising plant organ for future investigations. Future studies should focus on isolating and characterizing the active constituents, elucidating their mechanisms of action, and evaluating their efficacy and safety through in vivo models and clinical studies.

## Figures and Tables

**Table 1:** Quantification of phenolic acids and flavonoids by HPLC/DAD in the ethanolic extracts of leaves (EELSJ) and bark (EEBSJ) of *S. joazeiro* *.

Compounds	LD (mg/mL)	LQ (mg/mL)	EELSJ		EEBSJ	
			mg/g	%	mg/g	%
Caffeic acid	0.0001	0.0003	0.0456 ± 0.000057	0.00456	0.0470 ± 0.020785	0.0047
*p*-coumaric acid	0.0007	0.0025	0.0369 ± 0.017609	0.00369	-	-
Ferulic acid	0.0048	0.0162	0.2313 ± 0.002367	0.02313	0.150 ± 0.008660	0.0150
Cinnamic acid	0.0009	0.0032	-	-	-	-
Naringenin	0.0011	0.0036	-	-	-	-
Pinocembrin	0.0030	0.0102	0.3928 ± 0.007621	0.03928	-	-
Apigenin	0.0027	0.0092	-	-	-	-

* LQ: Limit of quantification, LD: Limit of detection, (-) unidentified. Results are expressed as mean (mg/g of sample) ± SD (n = 3).

**Table 2:** Leishmanicidal and cytotoxic activity of the ethanolic extract of leaves (EELSJ) and bark (EEBSJ) from *S. joazeiro*.

Compounds	IC_50_ (µg/mL)	CC_50_ (µg/mL)	SI
	Promastigote	J774.G8	
EELSJ	246.2 ± 0.089	343.3 ± 0.98	1.3943
EEBSJ	880.7 ± 0.062	5.866 ± 1.027	0.0066
Amphotericin B	0.54 ± 0.122	4.9 ± 1.216	90.7

(IC_50_): mean inhibitory concentration; (SI): selectivity index; (CC_50_): 50% cytotoxic concentration. Two-way ANOVA and Sidak’s multiple comparisons test.

## Data Availability

Data is contained within the article.

## Abbreviations

The following abbreviations are used in this manuscript:

ROS	reactive oxygen species
HPLC/DAD	high-performance liquid chromatography with a diode-array detector
IC50	mean inhibitory concentration
2-DR	Deoxyribose
SI	selectivity index
CC50	cytotoxic concentration
